# Embodied conversational agents for multimodal automated social skills training in people with autism spectrum disorders

**DOI:** 10.1371/journal.pone.0182151

**Published:** 2017-08-10

**Authors:** Hiroki Tanaka, Hideki Negoro, Hidemi Iwasaka, Satoshi Nakamura

**Affiliations:** 1 Graduate School of Information Science, Nara Institute of Science and Technology, Ikoma-shi, Nara, 630-0101, Japan; 2 Center for Special Needs Education, Nara University of Education, Nara-shi, Nara, 630-8538, Japan; 3 Developmental Center for Child and Adult, Shigisan Hospital, Ikoma-gun, Nara, 636-0815, Japan; Tokai University, JAPAN

## Abstract

Social skills training, performed by human trainers, is a well-established method for obtaining appropriate skills in social interaction. Previous work automated the process of social skills training by developing a dialogue system that teaches social communication skills through interaction with a computer avatar. Even though previous work that simulated social skills training only considered acoustic and linguistic information, human social skills trainers take into account visual and other non-verbal features. In this paper, we create and evaluate a social skills training system that closes this gap by considering the audiovisual features of the smiling ratio and the head pose (yaw and pitch). In addition, the previous system was only tested with graduate students; in this paper, we applied our system to children or young adults with autism spectrum disorders. For our experimental evaluation, we recruited 18 members from the general population and 10 people with autism spectrum disorders and gave them our proposed multimodal system to use. An experienced human social skills trainer rated the social skills of the users. We evaluated the system’s effectiveness by comparing pre- and post-training scores and identified significant improvement in their social skills using our proposed multimodal system. Computer-based social skills training is useful for people who experience social difficulties. Such a system can be used by teachers, therapists, and social skills trainers for rehabilitation and the supplemental use of human-based training anywhere and anytime.

## Introduction

Socialization and communication are critical factors that influence human social life. Persistent social skill deficits impede those with such afflictions from forming relationships or succeeding in social situations. An extreme example of people with social difficulties is those with autism spectrum disorders (ASD) [1]. Social skills training (SST), a general cognitive behavior therapy through which people with social difficulties can obtain appropriate social skills, is widely used by teachers, therapists, and trainers [2, 3]. Automating the SST process would simplify the acquisition of such social skills by those who require them.

It may also be easier for those with social communication difficulties to use computers than to directly interact with a human trainer [4]. Using computers in SST is motivated by the fact that even though people with social communication difficulties have difficulty during social interactions, they also show good or sometimes even superior “systemizing” skills [4]. Systemizing is the drive to analyze or build systems and understand and predict behavior in terms of underlying rules and regularities. The use of systematic computer-based training for people who need to improve their social skills can exploit the following facts: 1) such people favor computerized environments because they are predictable, consistent, and free from social demands; 2) they can work at their own speed and level of understanding; 3) training can be repeated over and over until the goal is achieved; and 4) interest and motivation can be maintained through computerized rewards [5–8]. Donna Williams, who has an ASD, explained her plight in school:

*“The comprehension of words works as a progression, depending on the amount of stress caused from fear and the stress of relating directly. At best, words are understood with meaning, as with the indirect teaching of facts by a teacher or, better still, a record, television, or book. In my first three years in the special class at primary school, the teacher often left the room and the pupils responded to the lessons broadcast through an overhead speaker. I remember responding to it without the distraction of coping with the teacher. In this sense, computers would probably be beneficial for autistic children once they had the skills to use one”*.

[9]

Previous works trained social skills using computers (see reviews in [10–13]), for instance, in the contexts of public speaking [14] and emotional regulation [8]. Another previous line of work addressed automated conversational coaches. Hoque *et al*. [15] proposed a dialogue system that trained people to improve their interview skills through real-time feature detection and feedback and achieved the following results: 1) a realistic task that trained actual users, 2) formative effective feedback that provided users with useful comments on particular behaviors that need improvement, and 3) the interpretation of user utterances to fuel the selection of backchannels or formative feedback. Even though these works included real-time or simultaneous feedback, most failed to follow the framework of medically evidenced SST, which consists of 1) instruction and target skills, 2) modeling, 3) role-playing, 4) feedback, and 5) homework.

We previously developed a system called an automated social skills trainer that completely adheres to SST’s basic training model through an embodied conversational agent [16]. Based on extracted audio features, this system provides feedback for improving users’ social skills. Experimental evaluation with graduate students showed that a larger training effect was found with our system than with control groups. Here, control groups received such traditional training as reading about social skills training and watching videos.

Since this previous work was just a first step, gaps obviously remain between human-based SST and automated social skills training. One gap is related to modality. Our previous work [16] considered only acoustic and linguistic features, and yet visual information (e.g., facial expression, head pose, and posture) is another essential feature of human-based SST [2, 3] and public speaking aids [17].

In this paper, we use our automated social skills trainer as a baseline and extend our system by adding visual information for more effective improvement in users’ social skills. The proposed system can be used not only by people who have difficulties in social interaction but also those with ASD who are its potential users [8]. This paper, which is an extension of a conference paper [18], is an experimental evaluation of people with ASD and provides a detailed multimodal system implementation.

The following summarizes this paper’s results:

1. We developed a system that follows traditional social skills training and integrates audiovisual features.
2. We confirmed the improvement of training effects by adding visual features.
3. We identified the maintainability of training effects by individuals with ASD.

In this paper, we describe the system implementation of the automated social skills trainer by referring to the basic human-based SST and report two experimental evaluations. In Experiment 1, we investigated the effect of multimodality in terms of a training effect. In Experiment 2, we applied a multimodal system to people with ASD and examined the training effect and relationships with other non-verbal behaviors.

## Basic SST and multimodal system implementation

The conventional SST is an established method that was originally developed to reduce anxiety and discomfort and obtain appropriate skills during social interaction [2]. SST effectively improves social skills for people with ASD [19].

SST can be classified as individual (one-to-one training) or group (one-to-many or many-to-many training) settings. One advantage of a group SST is that it enables participants to observe the behaviors of other participants and receive feedback. On the other hand, the advantage of an individual SST is that the training can be relaxed and simplified, and lessons can be tailored to individual needs.

The basic SST training model generally follows these steps: instruction, modeling, role-playing, feedback, and homework [3]. In this section, we describe them and our system implementation.

The automated social skills trainer was developed from MMDAgent (http://www.mmdagent.jp/), a Japanese spoken dialogue system that integrates speech recognition, dialogue management, text-to-speech, and behavior generation. MMDAgent works as a Windows application.

**Fig 1:**
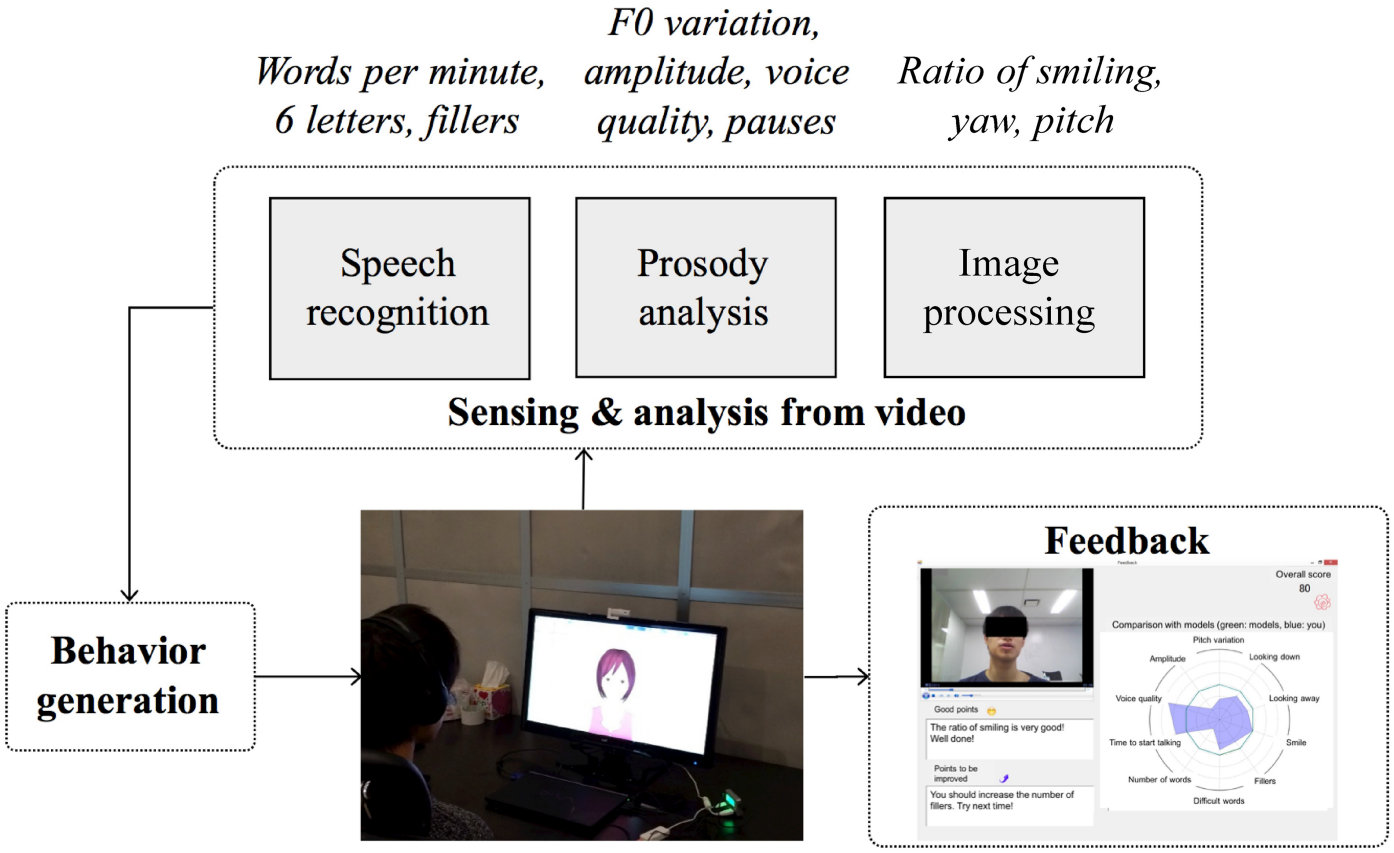
System framework of role-playing and feedback through interaction with computer avatar.

**Fig 2:**
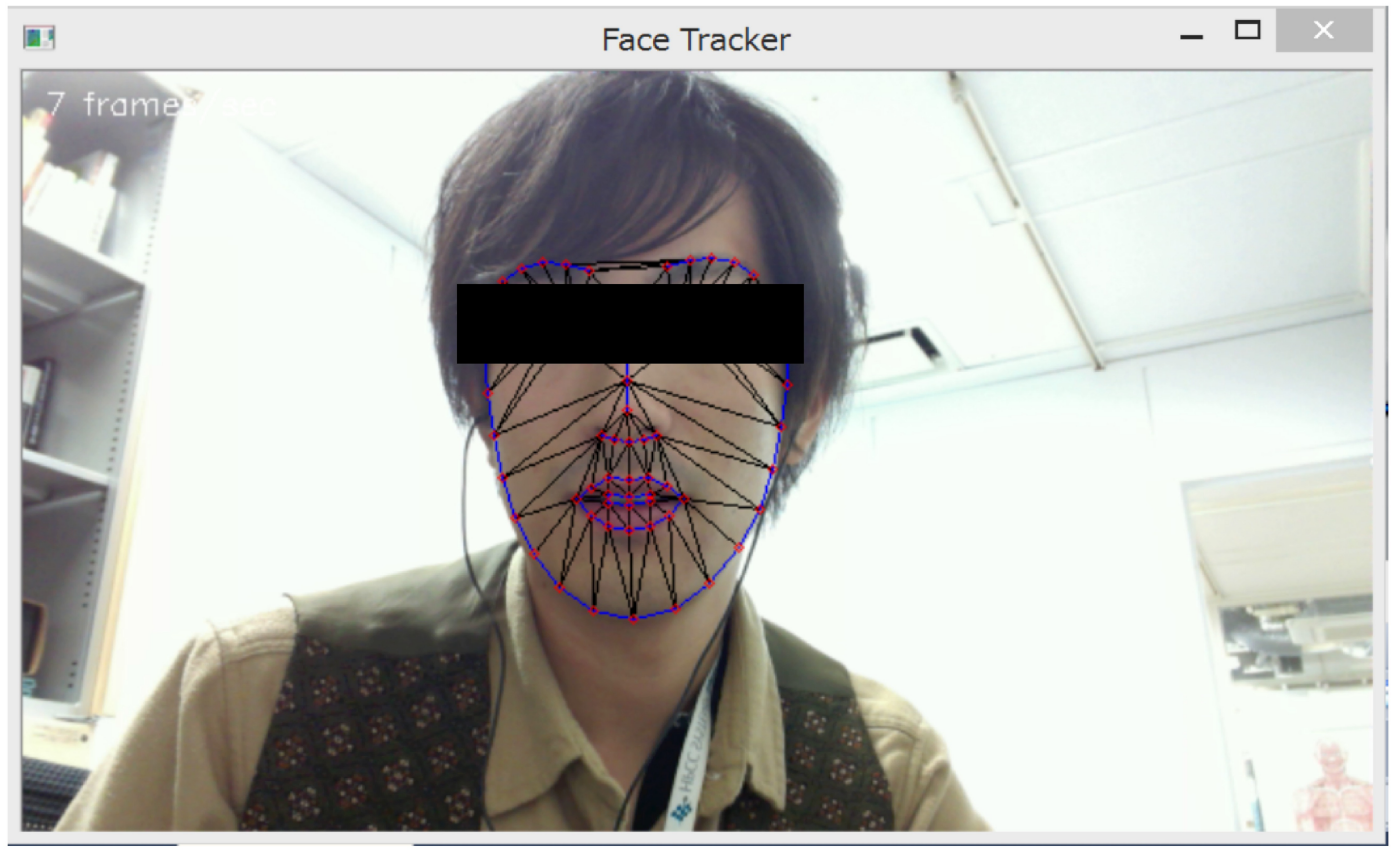
Extracted facial landmark points using face tracker.

**Fig 3:**
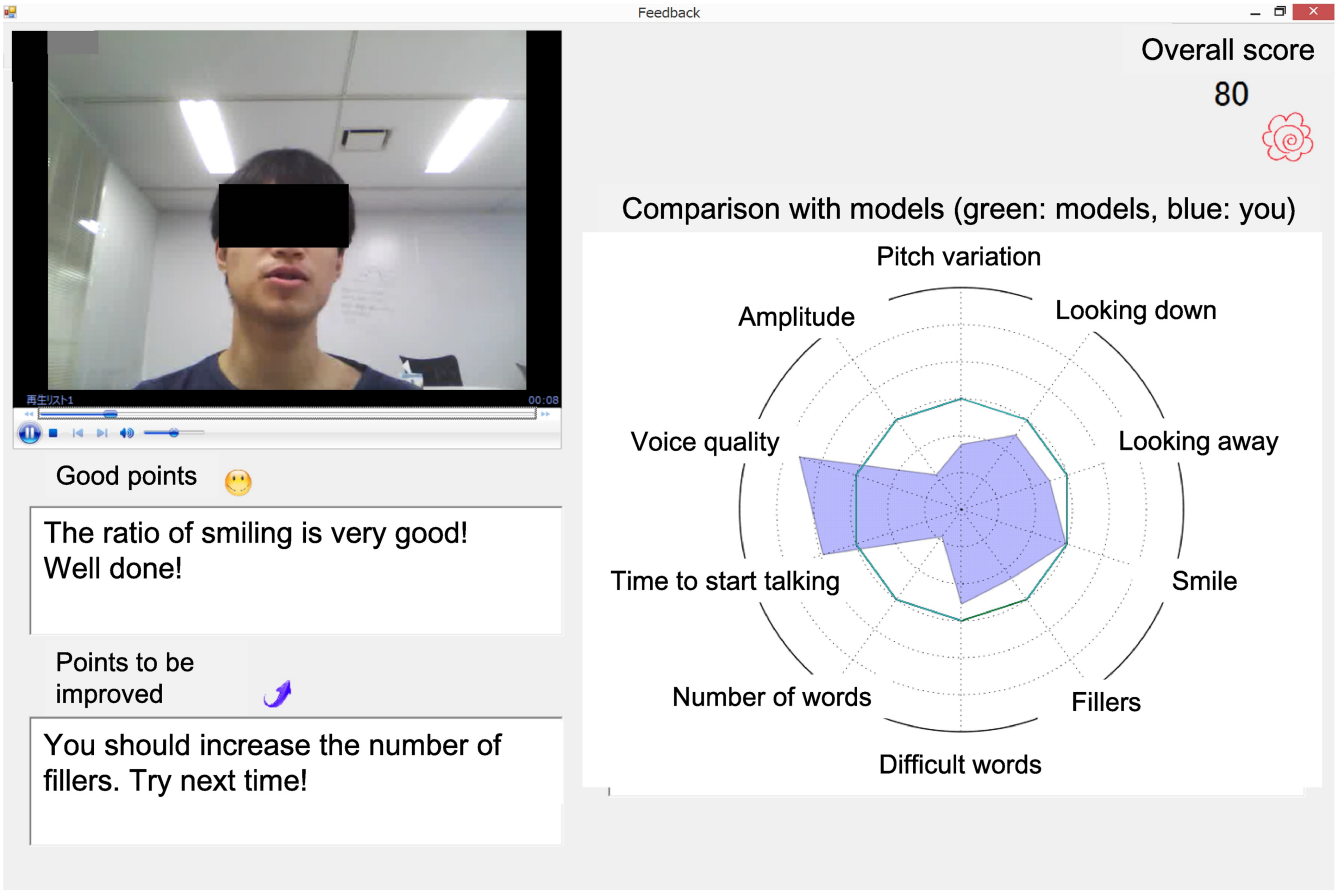
Audiovisual feedback provided by automated social skills trainer: User video, overall score, comparison with models, and positive comments.

1. **Instruction and target skills:** Instruction includes defining target skills and explaining their goals. After identifying the major social problems faced by the trainee, the skills to be learned are determined based on these problems.
  As an example of a target skill, the automatic social skills trainer sets a narrative, which emphasizes telling positive stories, because narrating stories is important for other higher-level skills. Other critical skills are listening to others, making requests, and expressing unpleasant feelings [3].
2. **Modeling:** Trainers act as a model and demonstrate the skill on which the users are focusing so that they can see what they need to do before attempting it themselves.
  The automated social skills trainer replicates this step for narrative skills by allowing users to watch a recorded model video of people with relatively good narrative skills. Users can watch and imitate such good examples.
3. **Role-playing:** Participants role-play their experiences for the trainer. This allows them to practice their own skills in the target situation. Trainers observe the participants’ social skills and focus on voice quality, amplitude, facial expressions, eye-gaze, and other non-verbal behaviors. Abnormal non-verbal behaviors in people with ASD have been reported [20–22].
  In the automated social skills trainer, the users also do role-plays. After the user says, “start the role-play”, the system says “please describe something fun you did recently.” The role-playing starts after the system’s request and continues for one minute. During this time, the avatar nods its head, and the system automatically senses and analyzes the audiovisual features from the user’s video (Fig 1).
  We extended this step by adding audiovisual information. To analyze the video’s information, we extracted a number of facial features using a constrained local model [23] based face tracker (Fig 2). The individual in this manuscript gave written informed consent (as outlined in the PLOS consent form) to publish these case details.
  Following Naim *et al*., [24], from a total of 66 feature points, we calculated the following features: the outer and inner eyebrow height, the outer and inner lip height, the eye opening, and the lip corner distance. Using these features, we modeled smiling faces with the Japanese Female Facial Expression database [25] that contains 213 images of seven facial expressions (six basic facial expressions and one neutral) performed by ten Japanese female models. Each image was rated with regards to six emotion adjectives by 60 Japanese subjects. In the database, we used 31 samples of happy faces and 30 samples of neutral faces and trained a model of two types of facial expressions using support vector machines with a linear kernel. For the video, we predicted whether the label belongs to the smiling or neutral class in each frame, and the proportion of the smiling frames among all the frames was called the smiling ratio. We verified that the model can be generalized to other speakers and video using the NOCOA+ database [26].
  In addition to the smile features, we separately incorporated two head pose features (yaw and pitch) based on the corresponding elements of the global transformations associated with rotation. The yaw indicates the horizontal direction, and the pitch indicates the vertical direction of the head pose. These features reflect looking away and looking down while talking [24]. We calculated the absolute value of the yaw and used the average of the entire frame to analyze the shift from the front. For the pitch, since both facing up (negative values) and facing down (positive values) are important, we calculated the average value of the entire video without taking absolute values. Because the system did not record images below the chest, we ignored non-facial gestures.
  Based on our previous work [16], we related the following extracted features to speech and language: F0 variation, amplitude, voice quality, pauses, words per minute, words over six letters, and fillers.
4. **Feedback:** Trainers provide feedback to help the participants identify their strengths and weaknesses.
  At the end of the role-playing, the automated social skills trainer immediately analyzes the features of the user’s video and determines its feedback, which it displays. Since displaying too many features in the feedback may confuse users, the system performs feature selection to identify effective features for defining narrative skills. This process was conducted in discussion with a professional social skills trainer.
  Based on the calculated features, the system displays feedback for the users (Fig 3). We simply display this feedback for greater comprehension and interpretation:
  - **User video:** Users can watch the recorded video and audio of their narrative.
  - **Overall score:** The system displays a predicted overall score, which motivates users to practice and raise their scores. We predict the overall scores using the generalized linear multiple regression method on a scale from 0 to 100 [16].
    Regarding the regression model’s features, because we analyzed the data and found that the smiling ratio for the model video was the highest, we added it to the regression model’s input features. We confirmed that the correlation coefficient between the predicted narrative skills and the subjectively evaluated skills [18] using leave-one-user-out cross validation was 0.55, which indicates a weak correlation, when using statistically significant features: words per minute, amplitude, words over six letters, and smiling ratio.
  - **Comparison with models:** The system uses a radar chart to compare the extracted features between the user’s current narrative and the model persons’ narratives in terms of a z-score, a statistical measurement of a score’s relationship to the mean in a group of scores. The users were informed to emulate the model in all aspects.
  - **Comments:** The system generates positive comments that reinforce the user’s motivation based on features whose values are the closest to those of the models. It also generates comments about points that need improvement based on a feature that has a median distance from the models. This choice of median (instead of the farthest) feature was based on discussions with a professional social skills trainer who noted that it might be fundamentally impossible for people with social communication difficulties to improve their worst points.
5. **Homework:** Trainers assigned relatively minor homework challenges that participants must complete on their own time throughout the week to facilitate the generalization of learned skills to daily life.
  The automated social skills trainer sets minor homework challenges that users must finish over the week. For example, the system informs users to tell a story to others throughout the week and will ask them to talk about that experience at the next session.

Using the automated social skills trainer, we performed two experimental evaluations to investigate the effect of feedback related to audiovisual information and its applicability to people with ASD.

## Experiment 1

In the first experiment, we examined the differences in the effectiveness of social skills training with feedback related to both audio and audiovisual features.

### Methods

#### Participants

We recruited 18 native Japanese-speaking graduate students (15 males and 3 females, ages 22 to 26) from the Nara Institute of Science and Technology.

#### Materials

We used the automated social skills trainer. We selected a single SST session and prepared our system on Microsoft Surface Pro 3. A webcam was placed on top of a laptop and a headset to record the video and audio of the participants.

#### Procedure

The Research Ethics Committee of Nara Institute of Science and Technology reviewed and approved this experiment (reference number 1309). Written informed consent was obtained from all the participants before the experiment.

Participants were given instructions by an experimenter and told that their speech and video would be recorded. The experiment was done in a soundproof room of the university, and participants were randomly separated into two groups: audio (six males and three females) and audiovisual (nine males). Even though the genders are not balanced, we performed another analysis without any female participants and found no effect on any of the statistical differences. All the participants first told a story to a known person (pre), used the automated social skills trainer for 50 minutes, and repeated their story to the same known person (post). In the training, the participants followed the procedure of the basic training model [3]. Because we did not control the video-watching or role-play durations, the participants could select the content by themselves (most repeated the modeling and role-play multiple times). The audio group received feedback regarding speech and language features [16], and the audiovisual group received feedback not only about the audio but also the smiling ratio, the yaw, and the pitch features.

A male social skills trainer, who has supervised young adult developmental support and performed SST for over three years, evaluated our participants’ overall narrative skills by Likert scores on a scale of 1 (not good) to 7 (good) [16]. He watched the randomly ordered pre- and post-video and rated the scores.

Before training, the initial scores of the two groups were not significantly different (p = 0.96 (two-tailed Student’s t-tests)); the audio group had a mean of 4.0 (sd: 0.91), and the audiovisual group had a mean of 4.2 (sd: 0.83).

We also performed the same evaluation with three more independent graduate student raters who have no experience with SST and randomly selected 25 samples from all of the video evaluated by the male social skills trainer. For each rater, each video was assigned to a class either above or below the average score for the rater, and we calculated the agreement between the classes. We confirmed that 96%, 72%, and 88% of the video samples agreed with the experienced social skills trainer, indicating good agreement.

#### Analysis

We calculated the pre- and post-scores to measure the training effect. We report the p-values of the Student’s t-tests (one-tailed) and the Cohen’s d values as a measure of the effect size.

### Results

**Fig 4:**
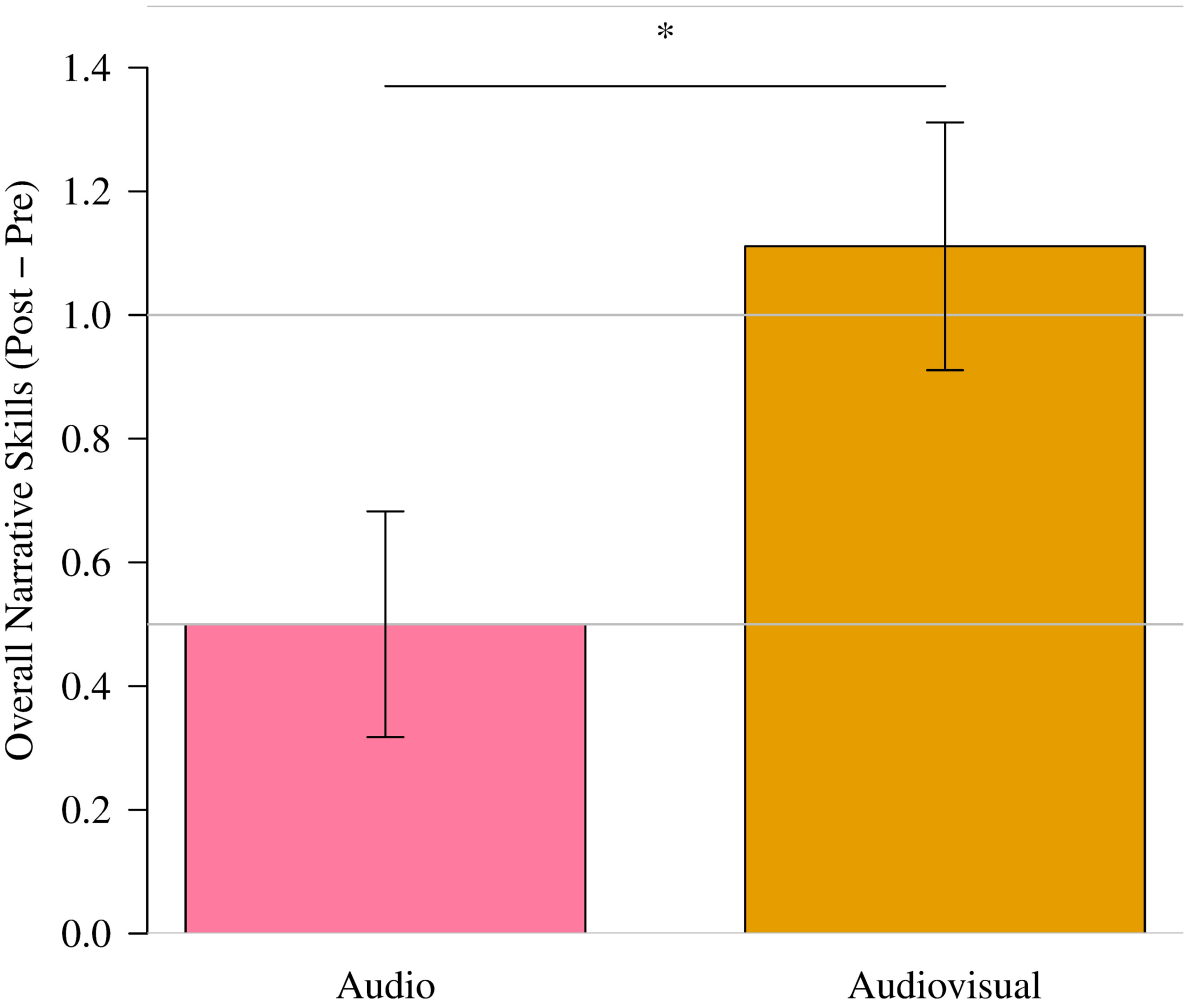
Improvement of overall narrative scores in audio and audiovisual groups. Error bars indicate standard error (*: p<.05).

Fig 4 shows the improvement of the overall narrative skills in the two groups. The audiovisual feedback led to a significant increase in the overall narrative skills (t(16) = -2.09, p = 0.03, d = 0.98).

### Discussion

The overall gain in skills using the audiovisual feedback was 1.1, which is comparable to or slightly greater than similar previous work: around a 1.0 point improvement through one-week interview skill training with a virtual tutoring agent [15] and around a 0.7 point improvement through narrative skill training [16]. In our previous analysis, the advantage of audiovisual feedback compared to audio feedback can probably be attributed to the slight improvements in the smiling ratio; the audiovisual group achieved a mean improvement and the audio group suffered a loss [18].

This result also reflects knowledge of human-based SST, which reflects the importance of smiling and facing directly ahead to express that the speaker is having fun [2].

## Experiment 2

In Experiment 2, we applied the automated social skills trainer to people with ASD and examined the training effect and the relationship to other non-verbal behaviors.

### Methods

#### Participants

For this experiment, we recruited from the Nara Autism Society, the Nara University of Education, and the Kyo Mental Clinic and accepted 12 applications, two of whom were removed because they did not complete every procedure of this experiment. The doctors or the therapists of the participants suggested that they attend this study based on the information described below. We recruited ten male participants (ages 7-19) who were diagnosed with one of the following: Pervasive Developmental Disorder-Not Otherwise Specified (PDD-NOS), High Functional Autism Spectrum Disorder, or Asperger Syndrome based on the DSM-IV-TR [27]. According to DSM-5 [1], which was published after these diagnostic procedures, all of these diagnoses fall within the ASD classification. The Wechsler Intelligence Scale for Children-Third Edition (WISC-IV) scores of the participants were all IQ > 70 [28]. All participants were native Japanese speakers. IDs were sorted by ages: ID 1: 7, ID 2: 7, ID 3: 9, ID 4: 11, ID 5: 12, ID 6: 12, ID 7: 12, ID 8: 13, ID 9: 16, and ID 10: 19.

#### Materials

We used the automated social skills trainer and selected a single SST session. We prepared the system on Microsoft Surface Pro 3. A webcam was placed on top of the laptop and the headset to record the audio and the video of the participants.

We slightly changed the system to reduce the number of features because displaying too many points might confuse people with ASD [29]. We finally selected the following five features: pitch, words per minute, amplitude, words over six letters, and smiling ratio [16, 18].

#### Procedure

Written informed consent was obtained from the parents of all of the participants before the experiment. We implemented an experiment with almost the same procedures as in Experiment 1. The participants first told a story to a stranger as pre (a), used the automated social skills trainer for 50 minutes, and finally repeated their story to the same stranger as post (b). In the training, the participants followed the basic training model’s procedure and received feedback regarding the five audiovisual features.

After the recording, we asked all of the participants to return and perform the post-recording again in three months (as follow-up). Unfortunately, only three participants returned and did so.

A female social skills trainer, who is also a licensed clinical psychologist and has supervised young children and performed SST for over three years, evaluated our participants’ overall narrative skills and their other non-verbal skills by Likert scores on a scale of 1 (not good/inappropriate) to 7 (good/appropriate) [17]. She randomly watched the (a) pre-, (b) post-, and follow-up videos and rated them.

#### Analysis

We used paired t-tests (one-tailed) and Cohen’s d values (only for overall narrative skills) to analyze the statistical differences between pre- and post-training. We also calculated the correlation coefficient with Person’s method to analyze the relationship between the overall narrative skills and other non-verbal behavior skills.

### Results

**Fig 5:**
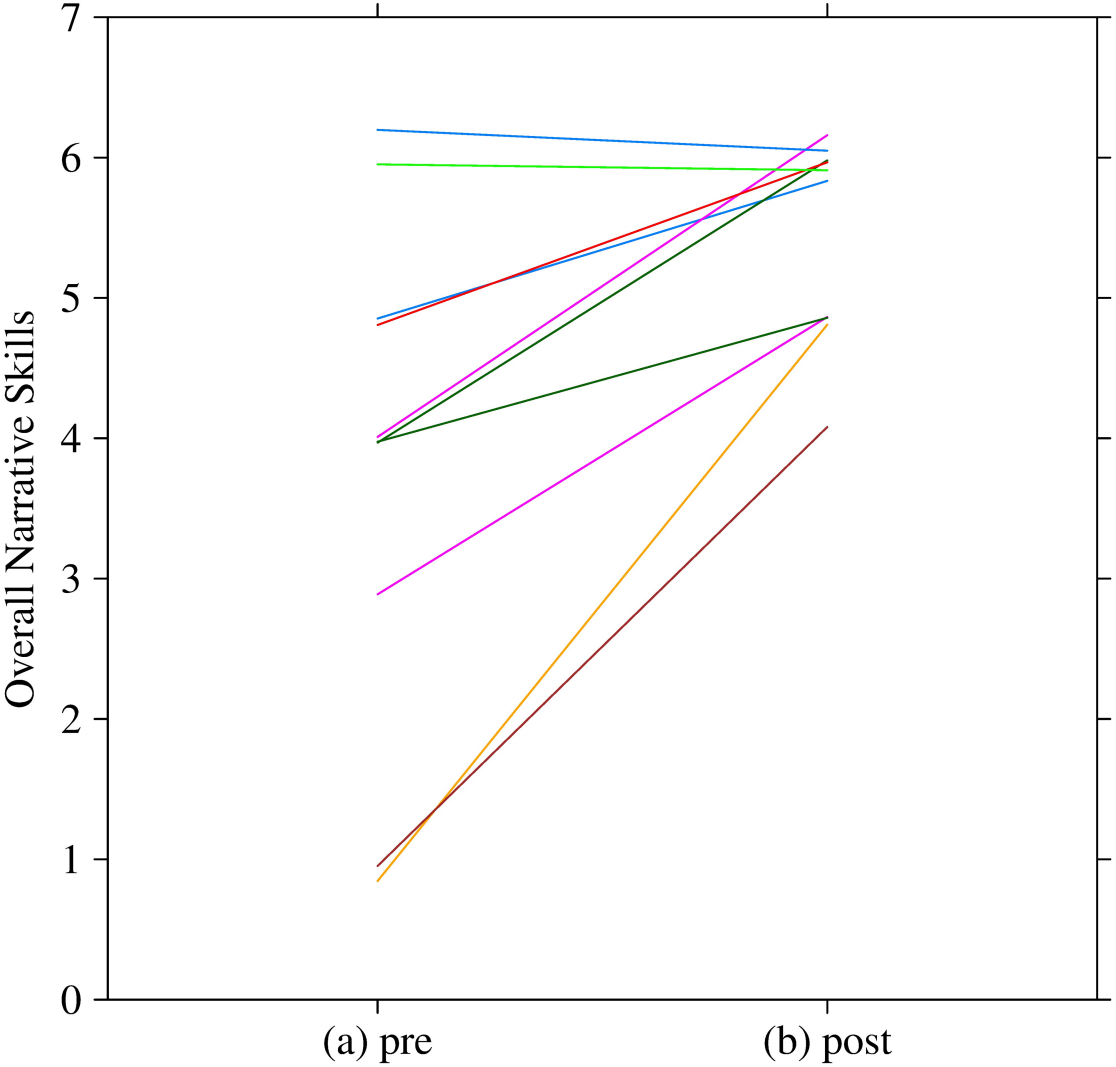
Overall narrative scores of pre- and post-training. Participants are indicated by color. We added a small amount of noise to separate identical points.

Fig 5 shows the pre- and post-scores for each participant. The system significantly increased their overall narrative skills (t(9) = -4.0, p = 0.003, d = 1.17). Table 1 indicates the individual improvements of the pre- and post-scores. The follow-up scores of the overall narrative skills were 6 (ID 3), 5 (ID 4), and 7 (ID 6).

**Table 1:** Relationship between participant ages and scores.

ID	Age	(a) Pre	(b) Post	(b) Post—(a) Pre
1	7	1	4	3
2	7	3	5	2
3	9	1	5	4
4	11	5	6	1
5	12	4	6	2
6	12	6	6	0
7	12	6	6	0
8	13	4	6	2
9	16	5	6	1
10	19	4	5	1

Table 2 shows the correlation coefficients between the overall narrative skills and other non-verbal behavior skills (all p < .05). We also confirmed the statistical difference between the pre- and post-training in these skills (except intonation skills) (p < .05).

**Table 2:** Correlation coefficient to overall narrative skills.

Non-verbal skill categories	Correlation coefficient
Structure	0.93
Speech rate	0.92
Gesture	0.90
Smoothness	0.89
Posture	0.85
Fillers	0.84
Intonation	0.81
Smile	0.80
Difficult words	0.80
Amplitude	0.79
Eye contact	0.78
Face orientation	0.73

### Discussion

We identified an increase in the overall narrative skills in most participants that was slightly greater than in Experiment 1. This indicates that the automated social skills trainer is useful and easy to understand for people with ASD and enhanced their narrative skills. Since our previous work [16] reported that improvements in overall narrative skills are correlated to the initial social skills of the users, this new result is consistent. This training effect doesn’t seem to be affected by any habituation effect because Hoque *et al*., found no large habituation effect in automated conversational coaching [15].

We identified a positive correlation between overall narrative skills and other non-verbal behaviors. This result indicates that the overall narrative skills were subjectively decided based on other non-verbal behavior skills [22]. Although the automated social skills trainer did not provide feedback regarding narrative structure or eye gaze, these elements were also improved after the training. Further analysis will scrutinize these relationships.

## General discussion

The focus of this study assessed the effectiveness of an automated social skills trainer with multimodal information that adheres to the basic human-based SST as closely as possible. We extended a previous method for automatic social skills training by adding audiovisual information regarding smiling ratio and head pose. To evaluate our proposed system’s effectiveness, we performed two experimental evaluations that examined the 1) advantages of using audiovisual features and 2) the training effect in children/young adults with ASD. For these experimental evaluations, we recruited 18 graduate students and ten people with ASD who used the proposed multimodal system. An experienced human social skills trainer rated the users’ overall narrative skills and the appropriateness of other non-verbal behaviors. Our results showed significant improvement in social skills between pre- and post-training as well as the relationships between the overall social skills and other non-verbal behaviors.

Previous work [16] found that automated social skills training provided a larger training effect than traditional training methods (e.g., reading about social skills training and video modeling [30]). In this study, we extended the automated social skills trainer by adding multimodal information that significantly improves the training effect (Experiment 1). This was also maintained in individuals with ASD (Experiment 2). Multimodal feedback is also useful for both members of the general population with social difficulties and people with ASD because it helps such people understand and improve their narrative skills, as was previously reported in human-based SST [2, 3]. This also indicates that audiovisual information is close to human-based SST and is effectively embodied in conversational agents. Since a previous work showed that improvement is related to initial social skills (people with lower social skills had greater training effects) [16], the results are consistent because people with ASD initially had lower social skills.

Even though this study was performed in Japanese with Japanese-speaking participants, the system’s language-dependent features are minimal, suggesting that it can be adapted to other languages. In particular, the system uses fixed utterances that are easily translated. However, because the features we extracted might be dependent on language or (more likely) culture, examining related behavioral features in other languages is an interesting avenue for future work.

Next we summarize the limitations of our paper. First, as a drawback, the current system did not consider the interactive aspects of dialogue; it targeted the teaching of narrative skills (a type of one-way storytelling) and used a simple strategy for non-verbal behavior generation. In future work, we will combine other interactive models such as nodding and blinking times and use not only a rule-based dialogue system but a more interactive conversation partner and generate simultaneous comments for feedback that was previously implemented [14].

Second, we performed our two experiments with an imbalanced male to female ratio (more male participants). Since gender has been identified as having a role in the training effect of conversational coaching (e.g., [15]) and is related to autism tendencies [31], investigating this effect in the context of automated social skills training is crucial for future work.

Third, the system did not attempt to comprehend the content of the user utterances. Although SST usually focuses on the non-verbal aspects of social interaction [3] and ignores the content of user utterances, the effectiveness of topic modeling in the context of job interview training has been shown [24]. We plan to consider the content of user utterances in future iterations of automated social skills training, although they will depend on the accuracy of speech recognition.

Fourth, even though we found that two people with ASD maintained their skills after three months, this result might just reflect the task’s repetition. We must consider the generalizability of learned skills to real situations by assessing quality of life, for example, [32].

Fifth, we did not consider the agent’s gender (we used only a female character). Since a previous study concluded that students perceive male agents as significantly more interesting, intelligent, useful, and lead to greater satisfaction than female agents [33], the issue of gender is critical for future work.

Last, in this paper we recruited participants with ASD without intellectual difficulties. Their doctors or therapists suggested that they participate, and we confirmed the following score: IQ > 70. However, because we did not obtain any actual IQ values, such mean values and standard deviations cannot be shown. This is a limitation because ASD includes a wide range (a spectrum) of symptoms, skills, and levels of disability, and the individual nature of ASD (e.g., intellectual ability and experiences with technology) is strongly associated with their characteristics. The participants of this study were only a small number of mild (high-functioning) cases, and it remains unclear whether all types of ASD have the same effect. We need to consider the relationship between the proposed system and the individual nature of ASD by obtaining actual IQ values and other relevant factors. Note that since this is a dialogue system that needs such users’ intellectual actions as conversation with agents, it might be difficult to use for people with other types of intellectual disabilities.

We reported that computer-based social skills training is widely useful for people with social difficulties to improve their narrative skills. Such a system can be used by SST teachers, therapists, and trainers for the rehabilitation and the supplemental use of human-based SST.

Future work will increase the number of ASD participants to examine the generalizability of this study. We also plan to add other target social skills to our automated social skills trainer based on a previous work [3] (e.g., listening skills) for comparisons with a human-based SST. We will scrutinize SST from the viewpoint of human-to-human and human-to-computer interactions, including types of agents and feedback [14]. In addition, we want to integrate multiple sessions with homework, which is done by human-based SSTs [2].
